# Vitamin D and kidney transplant outcomes: a protocol for a systematic review and meta-analysis

**DOI:** 10.1186/2046-4053-3-64

**Published:** 2014-06-14

**Authors:** Caitlin C Hesketh, Greg A Knoll, Amber O Molnar, Anne Tsampalieros, Deborah L Zimmerman

**Affiliations:** 1 Department of Medicine, University of Ottawa, Ottawa, ON, Canada; 2 Division of Nephrology, The Ottawa Hospital, Riverside Campus, 1967 Riverside Drive, K1H 7 W9 Ottawa, ON, Canada; 3 Clinical Epidemiology Program, Ottawa Hospital Research Institute, Ottawa, ON, Canada; 4 Department of Pediatrics, Children’s Hospital of Eastern Ontario, Division of Nephrology, Ottawa, ON, Canada

**Keywords:** vitamin D, 25-hydroxyvitamin D, 1,25-dihydroxyvitamin D, calcitriol, kidney transplantation, graft function, systematic review, protocol

## Abstract

**Background:**

Patients with end-stage renal disease who receive kidney transplants have improved survival and quality of life compared to patients on dialysis. Unfortunately, transplant patients often have a low vitamin D concentration, which has well-known effects on calcium and bone metabolism. The effect of vitamin D on other indicators of transplant function, such as glomerular filtration rate and acute rejection, remains unknown.

**Methods/design:**

We will conduct a systematic review of vitamin D status and outcomes after kidney transplantation. The primary objective is to assess the relationship between vitamin D and graft function using measured glomerular filtration rate (GFR) or estimated GFR from serum creatinine concentrations. Secondary outcomes will include acute rejection, chronic allograft nephropathy, proteinuria and graft loss. We will search MEDLINE, EMBASE, AMED and CINAHL for randomized and observational studies on adult renal transplant patients who received vitamin D supplementation or had serum vitamin D concentration measured. We will report study quality using the Cochrane Risk Assessment Tool for randomized controlled trials and the Newcastle–Ottawa Scale for observational studies. Quality across studies will be assessed using the GRADE approach. If pooling is deemed appropriate, we will perform meta-analyses using standard techniques for continuous and discrete variables, depending on the outcome. The results of this review may inform guideline development for vitamin D supplementation in renal transplant patients and highlight areas for further research.

**Systematic review registration:**

PROSPERO: CRD42013006464.

## Background

The prevalence of chronic kidney disease has been progressively increasing over the last two decades, with 40,385 Canadians being treated for end-stage renal disease in December 2011 [1]. Of these patients, 58% were receiving dialysis while 42% had a functioning renal transplant [1]. Studies have shown that patients who have received a kidney transplant have improved overall survival, less cardiovascular disease and improved quality of life compared to similar patients on dialysis [2]. Unfortunately, kidney transplants can still fail due to acute rejection and chronic allograft nephropathy; transplant failure can result in a threefold greater risk of death compared to patients with functioning grafts [3,4]. Given the increasing demand for renal transplants, it is important to identify modifiable risk factors implicated in graft failure to improve patient survival and quality of life.

Vitamin D is a steroid hormone involved in the regulation of calcium, phosphorus and bone metabolism. It is obtained from the diet and sun exposure and requires two hydroxylation steps for conversion to its physiologically active form, 1,25-dihydroxyvitamin D (calcitriol) [5]. The second hydroxylation step is performed by the enzyme 1α-hydroxylase, which is found predominantly in the kidney [5]. Many patients with a GFR less than 30 mL/min/1.73 m^2^ have an inadequate calcitriol concentration due to hyperphosphatemia-mediated inhibition of 1α-hydroxylase [6].

It is estimated that 17% of patients with chronic kidney disease have vitamin D insufficiency, which is defined as a vitamin D concentration between 15 and 30 ng/mL (40 to 70 nmol/L), and approximately 80% have vitamin D deficiency (vitamin D concentration <15 ng/mL or 37 nmol/L) [7]. Renal transplant patients are no exception. Stavroulopoulos *et al*. measured the vitamin D concentration for 104 patients within one year of a transplant and for 140 patients more than one year after a transplant [8]. They found that 97% and 94% of these patients, respectively, had vitamin D concentrations below 30 ng/mL [8]. Possible reasons for this include the intentional avoidance of sun exposure by patients on immunosuppressants and accelerated vitamin D catabolism by glucocorticoids [8]. Furthermore, there is a reluctance among physicians to prescribe vitamin D supplements for fear of precipitating hypercalcemia, hyperphosphatemia or hypoparathyroidism in patients with chronic kidney disease [9]. Aside from the general recommendations for those with chronic kidney disease, there are no specific guidelines for vitamin D supplementation for renal transplant patients.

Vitamin D deficiency may impact kidney allograft function, as vitamin D has known renoprotective properties. Vitamin D negatively regulates the renin-angiotensin-aldosterone system (RAAS) and has been shown to reduce RAAS-mediated renal fibrogenesis in a rat model of obstructive nephropathy [10,11]. Furthermore, vitamin D supplementation has been shown to decrease proteinuria in both human and animal models, which is a recognized risk factor for progressive renal failure. In rats, 1,25-dihydroxyvitamin D supplementation was found to reduce glomerulosclerosis and albuminuria through its anti-proliferative effects [12]. In humans, calcitriol and paricalcitol supplementation in addition to RAAS inhibition have been associated with significant proteinuria reductions in the settings of IgA nephropathy and diabetic nephropathy, respectively [13,14]. A significant association between vitamin D insufficiency and proteinuria has also been shown for renal transplant recipients; however, a causal relationship has not been established [15]. Considering these findings, RAAS inhibition and proteinuria reduction are two mechanisms by which vitamin D could positively affect graft function.

The immunomodulatory effects of vitamin D provide an additional basis for a role in renal transplantation. Calcitriol receptors are present on various immune cells, including T cells, B cells, monocytes and antigen-presenting cells [16]. In T cells, calcitriol suppresses helper T cell proliferation and differentiation, alters cytokine production and causes a shift from a pro-inflammatory Th1 response to a tolerogenic Th2 response [16,17]. Calcitriol also inhibits dendritic cell differentiation and maturation into antigen-presenting cells, which may be protective in transplantation [17]. In a rat model of chronic allograft nephropathy, administration of 1,25-dihydroxyvitamin D_3_ prolonged allograft survival, decreased episodes of acute rejection, reduced proteinuria and prevented histologic changes associated with chronic allograft nephropathy [18]. It follows that vitamin D supplementation may reduce acute rejection and chronic allograft nephropathy in humans through its interactions with the immune system.

Given the prevalence of vitamin D deficiency in patients with chronic kidney disease and the possible benefit of vitamin D in organ transplantation, it is important to establish recommendations to guide further therapy and research.

## Methods/design

### Research objectives

We will conduct a systematic review to determine the relationship between vitamin D concentration or supplementation and subsequent allograft function in kidney transplant recipients. The primary outcome will be graft function determined by the measurement of GFR (e.g. inulin clearance) or creatinine clearance (timed urine collection) as estimated from serum creatinine concentrations at different times post-transplantation. Secondary outcomes will include acute rejection, chronic allograft nephropathy, proteinuria and graft loss.

### Types of studies

In this review, we will include two types of study: (1) designs that examine the association between serum 25-hydroxyvitamin D or 1,25-dihydroxyvitamin D concentration and our stated primary and secondary outcomes and (2) designs that examine the effect of vitamin D supplementation on our stated outcome measures. Although this is the most inclusive approach, we anticipate several challenges. First, the thresholds used to define inadequate 25-hydroxyvitamin D levels in various studies may differ from recent guidelines [9]. For the purposes of this review, we will use thresholds selected by the study authors. Second, we anticipate heterogeneity in vitamin D formulation with some studies providing nutritional vitamin D supplements (e.g. cholecalciferol) and others using active vitamin D compounds (e.g. calcitriol and paricalcitol). As these formulations may affect graft function differently, pooling these results may be inappropriate (see Discussion).

### Search strategy

A comprehensive electronic search will be conducted using MEDLINE, EMBASE, AMED and CINAHL with the assistance of a librarian experienced in systematic reviews. A structured search strategy will be based on controlled vocabulary and relevant key terms and will be broad to prioritize sensitivity (see the Appendix). The references of included articles and existing reviews will be scanned for additional resources.

### Study screening and inclusion

All titles and abstracts from our comprehensive search will be screened by two independent reviewers. The inclusion and exclusion criteria used for each screening step are outlined below. If no abstract is available, the full text will be obtained unless the article can be confidently excluded by its title alone. In general, if there is any doubt as to whether a study should be excluded, the study will proceed to the full text screen to reduce the likelihood of incorrectly excluding a relevant study. Full text copies of potentially relevant papers will be obtained for independent analysis by two reviewers. Any disagreements will be reconciled by a third party.

### Inclusion criteria

Our review will focus on adult male and female subjects who received either a living donor or deceased donor kidney transplant. We will include retrospective and prospective studies (cross-sectional, case–control and cohort) and interventional studies (randomized and non-randomized). For studies that measure serum vitamin D concentration, we have not predefined when a sample needs to be measured (e.g. pre-transplant, 3-months post-transplant, 1-year post-transplant, etc.). For studies that assess the impact of vitamin D supplementation, all doses and formulations of vitamin D will be included. Studies must report one or more of the primary or secondary outcomes listed above to be eligible. Non-English articles will be included when there is a translator available at our institution. Publication dates will be restricted to 1990 and later given the changes in immunosuppression, transplant outcomes and measurement techniques for vitamin D that have occurred since then.

### Exclusion criteria

We will exclude case reports, narrative reviews, letters, animal studies and those with a sample size <30. Studies involving only pediatric patients or combined adult/pediatric populations where the data are not reported separately will be excluded. Studies involving multi-organ transplantation (e.g. kidney-pancreas) will also be excluded. We will exclude studies that report only bone-specific outcomes (e.g. fracture rates and bone mineral density), as there is an existing Cochrane review on this topic [19].

### Data extraction

Each study included in the review will undergo a standardized data extraction process using a pre-formatted spreadsheet. The extracted data will be verified by a second reviewer to reduce reviewer errors and bias. Information pertaining to study identification (first author, year of publication, number and location of centers), study design (type of study, sample size, inclusion and exclusion criteria, length of follow-up, type and dosage of vitamin D supplements) and patient population (age, gender, type of immunosuppression, donor type, duration of renal-replacement therapy, percentage of hemodialysis patients, time since transplant, number of prior transplants and cold ischemia time) will be included. These variables will be extracted for all types of studies.

The continuous variables extracted include GFR (as measured by inulin clearance), estimated GFR (eGFR; estimated from serum creatinine) and proteinuria. Proteinuria will be measured by 24-hour urine protein collection or the albumin-to-creatinine ratio, depending on the information that is available. For categorical variables, the extracted outcomes will be acute rejection, delayed graft function, graft loss and biopsy scores for interstitial fibrosis and tubular atrophy.

Time since transplantation will be recorded for each of the outcome variables. Time points will differ depending on which follow-up intervals were selected by the study author; this will be considered during data analysis. Outcomes will be recorded on separate spreadsheets for studies assessing serum vitamin D levels and studies assessing vitamin D supplementation (treatment group vs control).

### Quality assessment

If there are eligible randomized controlled trials, quality will be evaluated using the Cochrane Risk Assessment Tool. Studies will be assessed on randomization, generation of allocation sequence, allocation concealment, blinding and follow-up. We will evaluate observational studies with the Newcastle–Ottawa Scale. The quality of evidence across studies will be assessed for each outcome using the Grading of Recommendations, Assessment, Development and Evaluation (GRADE) approach. GRADE considers the risk of bias, consistency of results across studies, precision of the overall estimate across studies, magnitude of effect and importance of the outcome [20]. The quality of evidence will be rated as high, moderate, low or very low for each outcome.

### Analysis plan

For all included studies, we will provide a detailed description of the results in both tables and text. We will include data regarding study identification, study design and patient population (as outlined above). For studies assessing the effect of vitamin D supplementation, we will compare outcomes (e.g. acute rejection) for patients receiving vitamin D supplements to those for patients not receiving supplements.

For studies that measure serum vitamin D concentration, we will compare outcomes in patients with vitamin D insufficiency to patients with adequate serum vitamin D levels. As these definitions were not developed for the renal transplant population, we will also examine serum vitamin D as a continuous variable. We will pool treatment effect estimates where possible using standard statistical techniques. In the event that combining data across studies is not feasible due to inadequate information or excessive heterogeneity, we will use descriptive methods to present data by outcome.

Our inclusion criteria do not specify time points at which outcomes are measured (e.g. GFR at 12 months) to avoid excluding potentially useful information; however, we will account for timing during data analysis. We intend to group all studies together initially and then perform sensitivity analyses for different time points (e.g. GFR at less than one year or greater than one year), if possible.

## Discussion

In this systematic review, we will assess the impact of serum vitamin D concentration and vitamin D supplementation on renal transplant outcomes. While our methodology is designed to minimize selection bias, we do anticipate several limitations with this review: clinical and methodological heterogeneity, the quality of existing studies and the paucity of randomized controlled trials. Therefore, we anticipate that we may not be able to combine studies for a meta-analysis, but rather present findings using descriptive methods.

Our primary outcome is the GFR determined by direct measurement or estimation from serum creatinine concentrations. This outcome is subject to methodological heterogeneity, as the GFR is unlikely to be measured at the same time in all studies. We have not specified time points as inclusion criteria due to the risk of eliminating potentially relevant studies. Including studies with different time points may complicate data extraction and analysis; however, we feel it is important to increase the sensitivity of our review. If there are sufficient studies at a certain time point (e.g. 1-year post transplantation), we plan to pool the data where appropriate.

Another source of heterogeneity is the use of vitamin D compounds with different physiological mechanisms (e.g. cholecalciferol vs calcitriol vs paricalcitol), which may or may not have different effects on graft function. Previous data suggest that calcitriol may have greater parathyroid hormone (PTH)-lowering effects and a greater risk of hypercalcemia than nutritional compounds [21,22]. There may also be important differences between paricalcitol, a selective vitamin D receptor agonist, and the nonselective calcitriol regarding PTH-lowering, risk of hypercalcemia and overall survival [23,24]. We must consider that nutritional vitamin D and selective and non-selective vitamin D receptor agonists may affect transplant function differently, which may limit opportunities for pooling treatment effects. To maximize the yield of our literature search, however, we will include studies with all vitamin D formulations, including nutritional compounds, receptor agonists and vitamin D analogs. Including different formulations will increase the comprehensiveness of our review and may allow us to compare the effects of various vitamin D compounds.

Cross-sectional studies that compare serum vitamin D concentration with transplant outcome may provide important information; however, they also introduce the possibility of reverse causation. It has been well established that vitamin D levels decline with chronic kidney disease. Thus, in a cross-sectional study showing an association between low vitamin D concentration and reduced GFR, one cannot confidently exclude the possibility that reduced kidney function was the cause of vitamin D deficiency, rather than the result. Therefore, associations between serum vitamin D and GFR must be interpreted with caution.

Lastly, our results may be confounded by our inability to control for sun exposure and dietary vitamin D intake. Neither can be measured accurately in the typical outpatient setting. Studies that assess vitamin D supplementation without baseline serum concentrations are unlikely to capture a patient’s vitamin D status completely. Measuring baseline vitamin D concentrations will partially reduce this bias, though incompletely due to ongoing potential differences in vitamin D exposure via sunlight and diet. Although we recognize this as a limitation, after a renal transplant, patients are cautioned about sun exposure secondary to concerns about skin cancer. If patients follow this recommendation, differences in sun exposure should be minimized. The impact of nutritional vitamin D intake on serum vitamin D levels is unlikely to be significant.

In summary, our systematic review will provide insight into the effects of vitamin D insufficiency and/or replacement on renal transplant function. The results may have an immediate impact on patient care and facilitate guideline development for nutritional supplements for renal transplant patients. The data will also serve as the starting point for further research on vitamin D and kidney transplantation.

## Appendix: Search strategy

1. Kidney Transplantation/(80691)

2. ((kidney or renal) adj transplant$).tw. (58918)

3. 1 or 2 (90091)

4. exp Vitamin D/(44401)

5. vitamin d.tw. (38587)

6. Vitamin D Deficiency/(9201)

7. 25-hydroxyvitamin.tw. (7892)

8. Cholecalciferol$.tw. (1558)

9. Ergocalciferol$.tw. (416)

10. calcitriol.tw. (3875)

11. 1,25-dihydroxyvitamin D.tw. (4187)

12. (1,25$ adj OH2D).tw. (7)

13. or/4-12 (61278)

14. 3 and 13 (674)

15. limit 14 to english language (596)

16. animals/not humans/(3956436)

17. 15 not 16 (581)

18. remove duplicates from 17 (515)

## Abbreviations

eGFR: estimated glomerular filtration rate; GFR: glomerular filtration rate; GRADE: Grading of Recommendations, Assessment, Development and Evaluation; K/DOQI: Kidney Disease Outcomes Quality Initiative; PTH: parathyroid hormone; RAAS: renin-angiotensin-aldosterone system.

## Competing interests

The authors declare that they have no competing interests.

## Authors’ contributions

CH was involved in the design of the review, conducted the scoping searches, and drafted and revised the manuscript. GK conceived of the study, helped design the review, provided content expertise and revised the initial manuscript. AM was involved in the article screening process and quality assessment. AT was involved in the article screening process and quality assessment. DZ conceived of the study, helped design the review, provided content expertise, and revised the manuscript. All authors read and approved the final manuscript.
